# Healthcare workers’ knowledge and management skills of psychosocial and mental health needs and priorities of individuals with COVID-19

**DOI:** 10.1192/j.eurpsy.2022.367

**Published:** 2022-09-01

**Authors:** A. Hamdan-Mansour

**Affiliations:** The University of Jordan, School Of Nursing, Amman, Jordan

**Keywords:** mental health, Psychosocial Care, Covid-19, Mental Health Priorities

## Abstract

**Introduction:**

Individuals confirmed with COVID-19 were isolated or treated in medical and well-designated units; however, such a situation probably causing psychological and mental health problems that require prompt intervention.

**Objectives:**

The purpose of this study was to identify the knowledge and management of healthcare workers regarding psychosocial and mental health priorities and needs of individuals with COVID-19.

**Methods:**

This is a cross-sectional descriptive study. The data collected conveniently at one single point in time from 101 healthcare workers in Jordan directly managing the health of individuals with COVID-19.

**Results:**

healthcare workers have moderate to a high level of knowledge of psychological distress related to COVID-19; mean ranged from 50-70% agreement and confidence. Healthcare workers had moderate to a high level of management of psychosocial and mental health needs. In general, healthcare workers were able to identify mental and psychosocial health needs and priorities at a moderate level. Healthcare workers’ knowledge had a positive and significant correlation with age (r = .24, p = .012) and years of experience (r = .28, p =.004), and a significant difference was found in their management towards using mental and psychosocial care between those who are trained on psychological first aids and those who are not (t = - 3.11, p = .003).

**Conclusions:**

there is a need to train healthcare workers to integrate psychosocial and mental health care while managing psychological distress related to COVID-19.

**Disclosure:**

No significant relationships.

